# Complete Genome Sequences of Plasmid-Bearing Multidrug-Resistant *Campylobacter jejuni* and *Campylobacter coli* Strains with Type VI Secretion Systems, Isolated from Retail Turkey and Pork

**DOI:** 10.1128/genomeA.01360-17

**Published:** 2017-11-22

**Authors:** Daya Marasini, Mohamed K. Fakhr

**Affiliations:** Department of Biological Science, The University of Tulsa, Tulsa, Oklahoma, USA

## Abstract

We report the complete genome sequences of multidrug-resistant *Campylobacter jejuni* and *Campylobacter coli* isolated from retail turkey and pork, respectively. The chromosomes of these two isolates contained type VI secretion system genes. The two isolates also harbored large plasmids with antimicrobial resistance genes possibly contributing to their multidrug resistance.

## GENOME ANNOUNCEMENT

*Campylobacter jejuni* and *Campylobacter coli* are commensals in chicken and turkey but are known to cause gastroenteritis in humans. Compared to its prevalence in retail chicken, the prevalence of *Campylobacter* spp. is relatively lower in retail turkey but still significant (1, 2). The prevalence of *Campylobacter* spp. in retail pork samples is low compared to that of poultry (3). Multidrug resistance is more prevalent in *Campylobacter coli* than in *Campylobacter jejuni* (2–4). Multidrug resistance to more than seven antimicrobials was observed in *Campylobacter* strains isolated from turkey and pork (2, 3). Four *Campylobacter coli* pork isolates recovered in our laboratory were shown to be resistant to 16 antimicrobials (3). We have also previously reported that multidrug resistance was highly prevalent among *Staphylococcus aureus* strains isolated from turkey and pork (5, 6).

Here, we announce the complete genome sequences of multidrug-resistant *Campylobacter jejuni* YQ2210 and *Campylobacter coli* ZV1224, previously isolated in our laboratory from turkey and pork, respectively (2, 3). These two isolates were shown previously to carry large plasmids (7). A 72-h-old Mueller-Hinton (MH) blood broth culture was used to grow the bacteria for the isolation of genomic DNA using a Qiagen DNeasy blood and tissue kit (Qiagen, Valencia, CA). Plasmid DNA was isolated using the Qiagen plasmid midi kit (Qiagen). The paired-end sequencing of the Nextera XT library (Illumina, Inc., San Diego, CA) was performed by using an Illumina V2 reagent kit and run on an Illumina MiSeq desktop platform. The reagent kit was 2 × 150 cycles, which gave approximately 200× coverage of the sequenced genome. The output data were trimmed and *de novo* assembled using CLC Genomic Workbench 7.5.1 (Qiagen), and the resulted contigs were aligned against a reference genome using the CLC Microbial Genome Finishing Module (Qiagen).

The genome of the turkey isolate *Campylobacter jejuni* YQ2210 contained one full circular chromosome and two plasmids. The sizes of the chromosome, large plasmid pCJDM210L, and small plasmid pCJDM210S were 1,749,868, 44,808, and 5,170 bp, respectively. The chromosome contained 1,895 total genes, 1,851 coding sequences (CDS), and 1,760 coding genes with 44 RNAs and 91 pseudogenes. The larger plasmid contained 47 CDS and 1 pseudogene and harbored a type IV secretion system along with a tetracycline-resistant gene. The smaller plasmid contained 7 CDS with replication proteins and a few hypothetical protein genes.

The pork isolate *Campylobacter coli* ZV1224 contained one circular chromosome (1,837,306 bp) and two plasmids, pCCDM224L (55,234 bp) and pCCDM224S (32,270 bp). The chromosome contained a total of 2,013 genes, 1,966 CDS, and 1,898 coding genes with 47 RNAs and 68 pseudogenes. This genome also contained three noncoding RNAs. The large plasmid contained 61 CDS with multidrug-resistant genes and 3 pseudogenes. The smaller plasmid contained 39 CDS and 4 pseudogenes and showed the presence of some phage regulatory protein. Of special interest with regard to the two chromosomes was the presence of a type VI secretion system with homology to the one we previously reported to be present on megaplasmids (8).

### Accession number(s).

The genome sequences of the chromosomes and plasmids for the 2 strains are available on GenBank with accession numbers CP017859 (*Campylobacter jejuni* YQ2210 chromosome); CP017875 (*Campylobacter coli* ZV1224 chromosome); CP017857 and CP017858 (*Campylobacter jejuni* YQ2210 plasmids pCJDM210L and pCJDM210S, respectively); and CP017877 and CP017876 (*Campylobacter coli* ZV1224 plasmids pCCDM224L and pCCDM224S, respectively).
